# Drug resistance in protozoan blood parasites of livestock across the Middle East and North Africa

**DOI:** 10.4142/jvs.25357

**Published:** 2026-06-18

**Authors:** Bashir Salim, Abdullah Sheikh, Sara B. Mohammed, Saeed Alasmari

**Affiliations:** 1 Camel Research Center, King Faisal University, Al-Ahsa 31982, Saudi Arabia.; 2 Department of Microbiology, College of Veterinary Medicine, King Faisal University, Al-Ahsa 31982, Saudi Arabia.; 3 Department of Biology, Faculty of Sciences and Arts, Najran University, Najran 1988, Saudi Arabia.

**Keywords:** Antiprotozoal agents, one health, trypanosomiasis, babesiosis, theileriosis

## Abstract

**Importance:**

Protozoan blood parasites, including *Trypanosoma evansi*, *Theileria annulata*, and *Babesia* spp., remain major constraints to livestock health and productivity across the Middle East and North Africa (MENA). Disease control relies heavily on a limited number of chemotherapeutic agents that have been used for decades, raising concern about declining efficacy and emerging drug resistance.

**Observations:**

This review synthesized and critically evaluated field, experimental, and molecular evidence of antiparasitic drug resistance in major protozoan blood parasites of livestock in the MENA region. A narrative review was conducted using major scientific databases to identify peer-reviewed studies published between January 2000 and December 2025. Eligible studies reported treatment outcomes, experimental drug sensitivity, or molecular resistance markers in *T. evansi, T. annulata,* or *Babesia* spp. Evidence was organized by parasite species and evidence type; meta-analysis was not performed due to heterogeneity. The most consistent evidence of confirmed drug resistance was found for *T. annulata*, where buparvaquone treatment failure is repeatedly associated with mutations in the *cytochrome-b* and *TaPIN1* genes across multiple MENA countries. For trypanosomosis, recurrent field failures and experimental resistance to diminazene and isometamidium are reported, but validated molecular markers for *T. evansi* remain unavailable. In babesiosis, reported outcomes indicate variable or incomplete responses to imidocarb and diminazene, reflecting functional treatment failure rather than confirmed genetic resistance.

**Conclusions and Relevance:**

A clear mismatch exists between widespread field observations and limited molecular confirmation. Strengthened surveillance, standardized efficacy trials, rational drug use, and integrated parasite-control strategies are urgently needed in the MENA region.

## INTRODUCTION

Protozoan blood parasites remain among the most significant infectious diseases affecting livestock across the Middle East and North Africa (MENA), a region where animal agriculture plays a central role in food security, pastoral livelihoods, and rural economies [1]. High baseline exposure of livestock to hemoprotozoan parasites sustains continuous chemotherapeutic pressure in endemic areas. For example, in, Sudan, up to 20% of camels were infected with *Trypanosoma*, while cattle, sheep, goats, and equines were infected with *Theileria* and *Babesia* spp., illustrating the intensity of parasite transmission and the consequent reliance on repeated drug treatments in pastoral systems. Endemic diseases such as surra (*Trypanosoma evansi*), tropical theileriosis (*Theileria annulata*), and babesiosis (*Babesia* spp.) cause substantial morbidity and mortality in cattle, camels, sheep, goats, and equids, resulting in reduced productivity, high treatment costs, and major economic losses [2,3,4,5]. Reported prevalence of these protozoan infections across MENA varies widely depending on host species, ecology, and diagnostic method. Molecular and serological surveys indicate prevalence ranges of approximately 5%–30% for *T. evansi* in camels and mixed livestock systems, 10%–60% for *T. annulata* in cattle in endemic areas, and 5%–25% for *Babesia* spp. in cattle and equines, with higher values reported in intensively grazed and tick-infested systems [2,4].

In many MENA countries, these diseases are further amplified by distinctive ecological and production characteristics, including extensive grazing systems, transboundary livestock movements, mixed herding of multiple species, and climatic conditions that support the proliferation of vectors such as ticks and biting flies [6,7,8]. Ecological and production factors create persistent host-parasite-vector interfaces. This promotes continuous pathogen circulation and frequent reinfection across seasons and systems [4,9,10].

Historically, protozoan disease control in the MENA region has relied almost exclusively on a limited arsenal of chemotherapeutic agents. Drugs such as diminazene aceturate, isometamidium chloride, buparvaquone, and imidocarb dipropionate have remained the treatment backbone for four to six decades. During this period, very few compounds have been introduced into veterinary practice [11,12]. Diminazene aceturate, introduced in the 1950s, continues to be widely used against *T. evansi* and *Babesia* infections [13,14]. Buparvaquone, developed in the 1980s, remains the only highly effective drug for *T. annulata*. It is still used as the first-line therapy across many endemic countries [4,15]. Imidocarb dipropionate similarly persists as the primary therapeutic option for bovine babesiosis in most MENA livestock systems [3]. Heavy reliance on this aging chemotherapeutics creates strong selective pressure. This pressure is often exacerbated by administered without laboratory confirmation, appropriate dosing, or veterinary supervision [13,16]. At the global level, widespread resistance to diminazene aceturate and isometamidium chloride is now well documented in African animal trypanosomes. High failure rates are reported particularly for *Trypanosoma congolense*, with increasing evidence of cross-resistance between frontline trypanocides [17]. Although most molecular evidence currently originates from sub-Saharan Africa, the continued heavy reliance on the same drug classes suggests that similar resistance mechanisms may be emerging in MENA region but remain largely undocumented.

In recent years, field veterinarians, herders, and livestock producers across the region have increasingly reported cases of treatment failure and early relapse. These observations span a wide geographic range. They include camels with relapsing *T. evansi* infections in Sudan, Saudi Arabia, and Egypt. Additionally, cattle with tropical theileriosis are reportedly unresponsive to buparvaquone in Tunisia, Iran, Türkiye, and Iraq. Calves with babesiosis also show poor recovery after imidocarb therapy in Egypt and Sudan [18,19]. Such widespread clinical reports indicate that drug resistance may be far more prevalent than currently documented in scientific literature. Compared with helminths and ticks, the understanding of protozoan drug resistance in MENA remains limited and fragmented. This field lacks standardized surveillance tools. Under-detection is particularly pronounced in mobile pastoral settings where diagnoses are predominantly based on clinical signs. Furthermore, drug use in these areas is largely unsupervised. In South Kordofan State, Sudan, most livestock treatments are administered by herders without laboratory confirmation. This lack of veterinary oversight effectively masks the early emergence of drug resistance at the field level.

Among the protozoan parasites, *T. annulata* possesses the strongest molecular evidence of resistance. Studies from Tunisia, Sudan, Egypt, Iran, Türkiye, and Iraq have identified mutations in the *cytochrome-b* gene. These notably occur in Q01 and Q02 quinone-binding pockets. Mutations in the *TaPIN1* gene also confirm their association with buparvaquone failure [20,21,22,23,24]. These findings represent the most well-characterized examples of protozoan drug resistance in the region. They raise significant concerns as buparvaquone remains the only curative treatment for tropical theileriosis.

In contrast, drug resistance in *T. evansi* and *Babesia* spp. is far less understood. Field observations indicate diminishing effectiveness of diminazene, isometamidium, and imidocarb. However, molecular studies for these species remain scarce or nonexistent. For *T. evansi*, most evidence stems from clinical relapses and experimental infections showing reduced sensitivity [19]. No validated genetic markers are currently available for this species [19]. The situation for *Babesia*, is even more challenging. Despite widespread reports of treatment failure in cattle, sheep, and horses, no molecular markers have been confirmed in the MENA region.

These patterns highlight a major gap between practical experience and scientific understanding. While drug resistance is likely widespread, formal research remains limited and geographically uneven. Strengthening diagnostic capacity and developing regional molecular surveillance programs are essential to mitigate therapeutic failure.

**Fig. 1:**
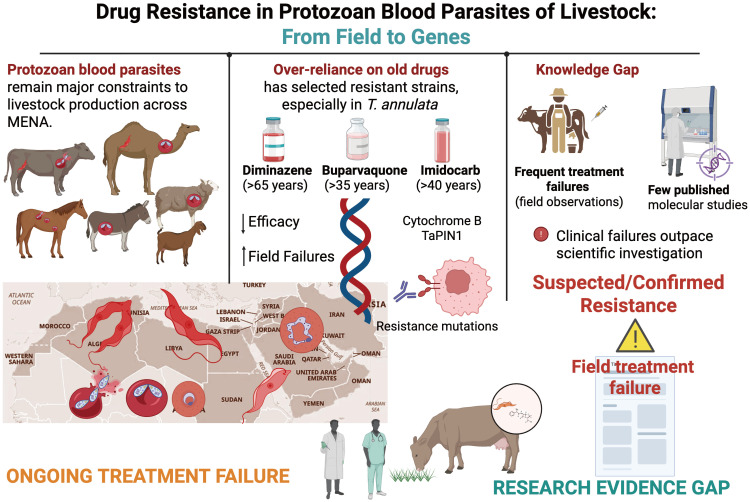
Drug resistance in livestock blood protozoan parasites in the MENA region: from field treatment failure to research evidence gaps. MENA, Middle East and North Africa; TaPIN1, T. annulata prolyl isomerase I gene.

This review consolidates current evidence on antiparasitic drug resistance in *Trypanosoma, Theileria,* and *Babesia* infections across the MENA region. By synthesizing clinical observations, molecular findings, and treatment-outcome data, we identify critical knowledge gaps. We also highlight emerging resistance trends and propose research priorities. These priorities aim to support sustainable parasite control and livestock productivity. The gap between field experience and scientific understanding is addressed by aligning reported treatment failures with experimental and molecular evidence. Where molecular confirmation is lacking, field observations are explicitly classified as phenotypic or functional evidence. This approach identifies priorities for future validation rather than overstating resistance. The overall structure of evidence, key drug classes, and major knowledge gaps addressed in this review are summarized in (Fig. 1).

## METHODS

### Literature search strategy

A comprehensive narrative literature review was conducted to identify studies reporting antiparasitic drug efficacy and resistance in protozoan blood parasites of livestock in the MENA region. Electronic database searches were performed using PubMed, Scopus, Web of Science, ScienceDirect, Google Scholar, and CAB Direct.

The primary search covered the period from January 2000 to December 2025. This period reflects the era during which most molecular and experimental resistance studies were published. Searches were conducted primarily in English. However, no strict language or date restrictions were applied due to the scarcity of regional resistance-focused literature. Relevant articles from regional journals were also identified through manual searches of reference lists and targeted journal websites. Search terms included combinations of parasite names such as *T. evansi, T. annulata,* and *Babesia* spp. Drug names used in the search included diminazene, isometamidium, buparvaquone, and imidocarb. Keywords included drug resistance, treatment failure, therapeutic efficacy, relapses, molecular markers, *cytochrome-b*, *TaPIN1*, and MENA or individual country names.

### Study selection criteria

#### Inclusion criteria comprised

- Peer-reviewed original research articles, short communications, and well-documented field or experimental studies.
- Studies reporting clinical treatment outcomes, experimental drug-efficacy data, or molecular evidence of resistance.
- Studies focusing on livestock or animal hosts infected with *T. evansi*, *T. annulata*, or *Babesia* spp.
- Studies conducted within the MENA region.
- Studies outside MENA were included if they provided relevant molecular or mechanistic context applicable to MENA parasites.

#### Exclusion criteria included

- Human-only studies.
- Studies lacking any therapeutic, experimental, or resistance-related data.
- Non-MENA studies that did not provide transferable molecular or methodological insights.
- Reviews without primary data, although key reviews were used for contextual framing.

### Data extraction and synthesis

Relevant information was extracted on parasite species, host species, and country. Data also included drug(s) evaluation, study design, treatment outcomes, and evidence type. Due to substantial heterogeneity among studies, a meta-analysis was not performed. These differences included host species, diagnostic methods, treatment protocols, and outcome measures.

Instead, findings were synthesized using a structured narrative approach, with evidence organized by 1) Parasite species (*Trypanosoma*, *Theileria*, *Babesia*), 2) Type of evidence (field observations, experimental studies, molecular investigations). This framework allowed clear differentiation between observational evidence, experimental confirmation, and molecularly validated resistance, addressing a key objective of the review. Treatment-success studies were included alongside reports of reduced efficacy to establish baseline drug performance and to contextualize suspected resistance.

### Quality considerations

Given the limited number of resistance-focused studies from the MENA region, no formal quality-scoring system was applied. However, priority was given to studies with clearly described methodologies, defined treatment regimen, appropriate diagnostic confirmation, and, where applicable, molecular validation. Limitations related to study design, sample size, or lack of molecular confirmation are explicitly acknowledged in the synthesis.

## OBSERVATIONS

### Evidence synthesis

#### Trypanosomosis (T. evansi and other Trypanozoon species)

##### 1) Field evidence

Field-based observations from several MENA countries, particularly Sudan, Egypt, and Saudi Arabia, describe recurrent treatment failure or relapse following administration of diminazene aceturate and isometamidium chloride, primarily in camels and cattle. Although precise prevalence estimates of treatment failure are rarely quantified, reports consistently describe herd-level recurrence affecting multiple animals within the same production unit rather than isolated cases.

In Sudan, the widespread and repeated use of diminazene aceturate and isometamidium chloride in camel herds and mixed livestock systems has been reported alongside high prevalence of *T. evansi, T. vivax*, and *T. congolense*. Cross-sectional surveys conducted in Blue Nile and West Kordofan States document continued transmission of multiple trypanosome species in cattle, sheep, goats, and camels despite routine trypanocide use, indicating that chemotherapy alone has not interrupted infection cycles and suggesting transient clinical improvement and post-treatment relapse under field conditions [25,26,27,28].

Comparable observations have been reported in Saudi Arabia, where local livestock systems rely heavily on diminazene-based treatment regimens [29]. Although most field reports remain descriptive and observational, their consistency across countries, host species, and production systems indicates reduced apparent drug efficacy under field conditions. These field-based observations are summarized in Table 1 [19,29,30,31,32].

**Table 1:** Trypanosomiasis: evidence for trypanocide resistance

No.	Species	Host	Country	Drug	Evidence type	Evidence strength	Key findings	Reference
1	*T. evansi*	Mice (experimental model)	Egypt	Quinapyramine, diminazene, melarsomine	Experimental	Confirmed (experimental)	Strong resistance to quinapyramine and diminazene, with relapses and mortality despite treatment; melarsomine achieved complete parasitological cure.	[19]
2	*T. evansi*, *T. vivax*	Rodent models	Sudan	Suramin, quinapyramine	Experimental	Confirmed (experimental)	Most isolates were resistant to suramin; quinapyramine sensitivity varied widely, with some isolates failing treatment in mice, while all remained susceptible to isometamidium.	[30]
3	*T. congolense*, *T. brucei*, and *T. vivax*	Cattle	Sudan	Diminazene aceturate and homidium bromide	Field/experimental	Confirmed (field + experimental)	Resistance to standard field doses of diminazene and homidium, with frequent post-treatment relapses; high-dose isometamidium achieved permanent cure in most strains, indicating multidrug-resistant bovine trypanosomes.	[31]
4	*T. evansi*	*In vitro* (rat models)	Sudan	Biquin, quinapyramine	Experimental	Confirmed (experimental)	Marked variability in quinapyramine efficacy by manufacturer; Tryquine cleared parasitemia, while Biquin and Nicholas Primal formulations failed to achieve cure.	[32]
5	*T. evansi*	Rats	Saudi Arabia	Diminazene	Field/experimental	Suspected reduced efficacy	Poor parasitological and immunological response to diminazene in a Saudi strain, with persistent infection despite treatment; no molecular confirmation available.	[29]

##### 2) Experimental evidence

Controlled experimental studies provide stronger evidence supporting reduced susceptibility of *T. evansi* to commonly used trypanocides. In Egypt, [19] experimentally infected Swiss albino mice with *T. evansi* and evaluated the efficacy of quinapyramine, diminazene aceturate, and melarsomine. The study demonstrated early relapse, sustained parasitemia, and high mortality following treatment with quinapyramine and diminazene aceturate, even at increased doses. In contrast, melarsomine administered at 0.5 mg/kg achieved complete parasitological clearance and prevented relapses during the follow-up period [19].

These experimental findings provide direct evidence of reduced drug susceptibility in *T. evansi* isolates and support the field observations of poor response to widely used trypanocides in parts of the MENA region. Key experimental outcomes are presented in Table 1.

##### 3) Molecular evidence and gaps

Despite consistent clinical and experimental indications of reduced trypanocide efficacy, molecular evidence of resistance in *T. evansi* from the MENA region remains very limited. A global review of drug resistance in blood parasites of cattle highlights that resistance mechanisms are relatively well characterized in African trypanosomes such as *T. brucei* and *T. congolense*, including defects in drug transporters and altered drug uptake pathways; however, comparable molecular markers have not been validated for *T. evansi* [33].

To date, no study from the MENA region has conclusively linked specific genetic mutations, transporter defects, or molecular alterations in *T. evansi* to documented field treatment failure. Available evidence remains predominantly phenotypic, based on clinical relapse, reduced survival benefit, and experimental treatment outcomes, rather than genotypic confirmation. This discrepancy between consistent field and experimental observations and the absence of validated molecular markers represents a major knowledge gap, as summarized in Table 1 (Trypanosomosis: evidence type field/experimental versus molecular).

#### Theileriosis (T. annulata)

##### 1) Field evidence

Field-level evidence of reduced buparvaquone efficacy in *T. annulata* has been reported repeatedly across MENA region. A recent systematic review by [34] identified Tunisia, Türkiye, Egypt, Sudan, Iran, Pakistan, and Iraq as primary sources of confirmed or suspected resistance. These findings highligh *T. annulata* as the protozoan parasite with the strongest regional evidence base for drug resistance.

The earliest influential field report originated from Tunisia [35]. This documented fatal tropical theileriosis in cattle despite repeated buparvaquone treatment. Subsequent investigations in Sudan, Egypt, Iran, and Iraq reported similar treatment failures. These were typically characterized by persistent parasitemia, lack of clinical improvement, or mortality. In several studies, non-responsive animals were compared directly with successfully treated controls. This strengthening the inference that treatment failure was not solely due to management or dosing errors (Table 2) [18,21,22,24,34,35].

**Table 2:** Evidence of buparvaquone resistance in *T. annulata* in Middle East and North Africa

No.		Species	Host	Country	Evidence type	Key findings	Reference
Field evidence							
	1	*Theileria spp*.	Cattle	Multi-country (Tunisia, Türkiye, Egypt, Sudan, Iran, Pakistan, China, Germany)	Systematic review	Cytochrome-b (Q01/Q02) and TaPIN1 mutations repeatedly associated with buparvaquone resistance; proposed molecular biomarkers	[34]
	2	*T. annulata*	Cattle	Tunisia	Field report	First confirmed buparvaquone-resistant outbreak; 4/7 cattle died despite repeated treatment	[35]
	3	*T. annulata*	Cattle	Egypt	Field + molecular	Non-responding animals carried cyt b mutations at codons 253 and 262 (Q02); responsive isolate showed Q01 mutation only	[18]
	4	*T. annulata*	Cattle	Sudan	Molecular (field isolates)	Non-synonymous cyt b mutations detected near Q01/Q02 drug-binding regions	[21]
	5	*T. annulata*	Cattle	Iran	Molecular (field isolates)	S109G and P233S mutations in cyt b Q(o) sites associated with fatal treatment failure	[22]
	6	*T. annulata*	Buffalo	Iraq	Molecular (field isolates)	Genetic alterations in cyt b drug-binding codons; Iraqi isolates distinct from regional strains	[24]
Experimental and molecular evidence							
	7	*T. annulata*	*In vitro* (clonal lines)	Tunisia	Molecular	Five cyt b mutations identified; two Q02 mutations present in 68% of resistant clones	[20]
	8	*T. annulata*	Cattle-derived cell lines	Türkiye	Molecular	Six non-synonymous and six synonymous cyt b mutations; V135A and P253S implicated; TaPIN1 A53P absent	[23]
	9	*T. annulata*	*In vitro* macrophage lines	-	Experimental resistance induction	Stepwise drug exposure produced resistant lines with increased IC₅₀ and cyt b mutation M128I	[37]
	10	*T. annulata*	Cattle	Sudan	Molecular	*TaPIN1* *A53P* mutation detected; frequent co-occurrence with cyt b mutations supports dual-marker resistance model	[41]
	11	*T. annulata*	Cattle & buffalo (field + cell lines)	Pakistan, Tunisia, Türkiye	Molecular	Mutations 129G (Q01) and 253S/262S (Q02) detected in both resistant laboratory lines and field isolates	[40]

cyt b, cytochrome-b.

An epidemiological study from Egypt further demonstrated that irregular antiprotozoal drugs use strongly predicts of *T. annulata* infection at the animal level [36]. While buparvaquone susceptibility was not directly assessed, the finding provides indirect evidence. It suggests improper chemotherapy practices may contribute to the selection of less susceptible parasite populations.

##### 2) Experimental evidence

Experimental and semi-controlled investigations provide strong support for these field observations. In Sudan, investigators [21], examined cattle with poor response to buparvaquone. They demonstrated that non-responsive animals harbored *T. annulata* isolates with specific mutations in the *cytochrome-b* gene. Similarly, in Egypt, [18] analyzed one buparvaquone-responsive and five non-responsive cattle. The results showed that mutations at codons 253 and 262 within the Q02 drug-binding site were consistently associated with treatment failure. Conversely, responsive isolates carried different substitutions in the Q01 region.

Experimental confirmation of resistance has also been achieved using in vitro and cell-line systems. A study in Tunisia [20] analyzed 28 cloned *T. annulata* lines from Tunisia and reported that resistant clones carried a higher frequency of nonsynonymous *cytochrome-b* mutations, particularly within the Q02 region. More recently, generated buparvaquone-resistant parasites through stepwise drug exposure in transformed macrophages [37], demonstrating a significant increase in IC_50_ values and identifying a shared M128I mutation in *cytochrome-b*. Collectively, these studies provide reproducible experimental evidence that buparvaquone resistance can arise under drug pressure and is associated with specific genetic changes.

##### 3) Molecular gaps

Molecular investigations have identified *cytochrome-b* as the primary genetic target associated with buparvaquone resistance in *T. annulata*. Across studies from Tunisia, Sudan, Egypt, Iran, Türkiye, Iraq, and Pakistan, recurrent nonsynonymous substitutions have been reported. These occur in the Q01 and Q02 quinone-binding pockets. Standardized notations for these mutations include Met129Gly, Pro253Ser, and Ser262Gly [36,38,39]. The recurrence of these mutations across multiple countries suggests parallel selection under drug pressure. Alternatively, it may indicate regional dissemination of resistant genotypes (Table 2) [20,23,37,40,41].

In addition to *cytochrome-b*, *TaPIN1* has emerged as a second resistance-associated marker. Researchers [35] identified an alanine-to-proline substitution (Ala53Pro) within the catalytic loop of *TaPIN1* in Sudanese isolates associated with treatment failure. Many of these isolates also carried *cytochrome-b* mutations. This supports a dual-marker resistance model. Follow-up studies from Egypt reported concurrent variation in both *cytochrome-b* and *TaPIN1* loci [36], reinforcing this association.

Despite the strength of molecular evidence for *T. annulata*, important gaps remain. Most studies are geographically localized and based on limited sample sizes. The true regional prevalence of resistant genotypes remains unknown. Furthermore, genotype–phenotype relationships have not been systematically quantified under controlled infection conditions. This includes the relative contribution of *cytochrome-b* versus *TaPIN1* mutations. Nevertheless, *T. annulata* remains the only protozoan parasite in the MENA region with consistent, convergent evidence of drug resistance.

#### Babesiosis (Babesia spp.)

##### 1) Field evidence

Field and clinical reports from several MENA countries indicate variable therapeutic responses to imidocarb dipropionate and diminazene aceturate. These observations span bovine, ovine, equine, and canine babesiosis. However, unlike theileriosis, these findings remain largely observational and non-confirmatory.

In Egypt, epidemiological studies found that irregular antiprotozoal drug use is a significant risk factor for *Babesia* infections. This highlights how treatment practices sustain transmission rather than demonstrating resistance *per se* [42]. While imidocarb generally improves clinical signs in infected cattle, parasitemia occasionally persists. This indicates variable therapeutic performance rather than confirmed resistance. These livestock treatment outcomes are summarized in Table 3 [42,43,44].

**Table 3:** Babesiosis (*Babesia* spp.) in Middle East and North Africa livestock: reported treatment outcomes (no confirmed molecular resistance)

No.		Species	Host	Country	Drug(s)	Evidence Type	Outcome	Reference
Reported treatment outcomes								
	1	*B. caballi*	Horses	Saudi Arabia	Imidocarb	Field	Clinical recovery with variable hematological response	[42]
Experimental evidence (contextual only)								
	2	*Babesia* spp.	Mice	Japan/Egypt	Diminazene + pyronaridine	Experimental	Combination superior to monotherapy	[43]
	3	*B. negevi*	Dogs	Israel	Imidocarb; atovaquone–azithromycin	Clinical	Imidocarb failed to clear parasitemia; PCR-negative only after combination therapy	[44]

Evidence from Sudan remains largely anecdotal. Veterinary reports describe cattle with delayed or incomplete clinical recovery following standard doses of imidocarb or diminazene. Since these lack support from controlled trials or molecular studies, they should be interpreted as suspected reduced efficacy.

In Israel, dogs naturally infected with *Babesia negevi* showed clinical improvement after imidocarb administration. However, these animals remained polymerase chain reaction (PCR)-positive for up to seven months. Complete parasite clearance was achieved only after treatment with atovaquone–azithromycin. This outcome indicates functional treatment failure without molecular confirmation of resistance [44]. This example of functional treatment limitation is presented in Table 3. Collectively, these findings point to heterogeneous clinical responses across host species and settings.

##### 2) Experimental evidence

Experimental data regarding babesiosis remain limited in the MENA region. However, indirect evidence suggests that classical monotherapies may be losing relative effectiveness. A recent study demonstrated that combining pyronaridine tetraphosphate with diminazene aceturate suppressed *Babesia* growth more effectively than high dose diminazene alone [45]. This experimental evidence is also included in Table 3. This supports the concept that combination therapy may outperform traditional monotherapy when drug performance is suboptimal. While this study does not demonstrate resistance, it supports concerns regarding the adequacy of diminazene monotherapy. Outside food-producing animals, experimental follow-up in canine babesiosis further illustrates incomplete drug performance. In *B. negevi*–infected dogs, repeated imidocarb treatment reduced parasitemia but failed to achieve molecular clearance [44]. This reinforces the distinction between clinical improvement and parasitological cure.

##### 3) Molecular gaps

Despite recurring reports of inconsistent therapeutic outcomes, no molecular markers of imidocarb or diminazene resistance have been documented in *Babesia* spp. from the MENA region. None of the available studies from Egypt, Sudan, Saudi Arabia, or North Africa have investigated mutations in known or putative drug-target genes, including *Babesia cytochrome-b*, apicoplast-associated loci, or drug transporter genes.

This limitation is not unique to MENA. Global reviews highlight that, unlike *T. annulata* and African trypanosomes, resistance mechanisms in *Babesia* spp. remain poorly characterized worldwide, with no validated molecular markers currently available for routine surveillance [2,17]. Consequently, assessments of drug performance rely almost exclusively on clinical response, a method with low specificity and high susceptibility to confounding factors such as delayed diagnosis, high parasitemia, inconsistent dosing, poor-quality formulations, or mixed infections.

The complete absence of molecular validation represents a critical gap in the evidence base for babesiosis in the MENA region. Given the long-term and intensive use of imidocarb dipropionate as the primary treatment for *B. bovis*, *B. bigemina*, and *B. caballi*, the emergence of resistance could occur undetected unless targeted molecular surveillance, controlled efficacy trials, and post-treatment PCR monitoring are implemented.

### Cross-cutting themes

#### Treatment success baseline

Establishing a baseline of treatment success is essential for providing an interpretative framework for suspected drug resistance. Without clearly documented examples of expected therapeutic efficacy, it is difficult to determine if observed failures represent genuine reductions in drug sensitivity or arise from non-genetic confounders. Several studies from the MENA region report satisfactory therapeutic outcomes following administration of imidocarb dipropionate or diminazene aceturate in cattle, equines, and other livestock. These include controlled treatment trials in Egypt, therapeutic investigations in Arabian horses in Saudi Arabia, and earlier field studies from Sudan demonstrating favorable clinical and hematobiochemical responses following conventional treatment protocols. Key treatment success is presented in Table 4 [19,29,41,42,43,45,46,47,48,49,50].

**Table 4:** Documented treatment successes

No.	Parasite/disease	Host	Country	Drug/regimen	Study type	Outcome	Reference
1	*T. annulata*	Cattle	Iran	Parvaquone vs buparvaquone	Field trial	High recovery; buparvaquone superior	[45]
2	Tropical theileriosis	Cattle	Türkiye	Buparvaquone	Field trial	High cure rate	[46]
3	Tropical theileriosis	Crossbred calves	Egypt	Buparvaquone	Field trial	Clinical recovery	[47]
4	Theileriosis (*T. annulata*)	Pregnant cows	Sudan	Buparvaquone	Field study	High cure rate	[48]
5	Tropical theileriosis	Buffalo	Egypt	Buparvaquone	Clinical study	Clinical recovery	[49]
6	Bovine babesiosis	Cattle	Egypt	Imidocarb dipropionate	Clinical trial	Effective parasite clearance and clinical improvement	[41]
7	*B. caballi*	Horses	Saudi Arabia	Imidocarb dipropionate	Clinical study	Resolution of clinical signs	[42]
8	*B. ovis*	Sheep	Türkiye	Imidocarb dipropionate	Experimental	Highly effective	[50]
9	*Babesia* spp. (*B. bovis*, *B. equi*, *B. microti*)	In vitro & mice	Japan/Egypt collaboration	Pyronaridine + Diminazene	Experimental	Combination therapy synergistic or additive; superior to high-dose diminazene alone; improved hematologic recovery	[43]
10	Surra (*T. evansi*)	Mice (camel strain)	Saudi Arabia	Diminazene, suramin, quinapyramine	Experimental	Reduction in parasitemia following treatment	[29]
11	Surra (*T. evansi*)	Swiss mice	Egypt	Melarsomine vs. diminazene	Experimental	Melarsomine fully effective	[19]

This table summarizes studies reporting successful therapeutic outcomes following treatment with standard antiprotozoal drugs. These data provide a baseline reference for expected drug performance and are included to facilitate comparison with reports of treatment failure or suspected reduced efficacy presented elsewhere in the manuscript. Inclusion in this table does not imply absence of resistance in the region but reflects documented clinical or experimental treatment success under defined conditions.

These studies provide reference benchmarks for normal drug performance under field conditions. Documented treatment success highlights that imidocarb and diminazene remain effective in many settings across the region, reinforcing the heterogeneity of therapeutic outcomes observed in MENA livestock systems. The presence of both successful and inconsistent responses emphasizes that poor treatment outcomes cannot be automatically attributed to drug resistance. Factors such as delayed diagnosis, high parasitemia at presentation, inaccurate dosing, compromised drug quality, reinfection, or concurrent hemoparasitic infections may strongly influence clinical response.

#### Drug use practices and selection pressure

A consistent theme across parasites and countries in the MENA region is the heavy reliance on a small number of aging chemotherapeutic agents and the widespread use of empirical treatment practices. In many pastoral and semi-nomadic systems, livestock owners rely on symptom-based diagnosis and frequently administer veterinary drugs without laboratory confirmation or professional supervision. Owners also often combine modern chemotherapeutics with traditional remedies. Such practices create intense and continuous selection pressure favoring the emergence and spread of less susceptible protozoan parasite populations. Across the region, treatment of protozoan blood parasites depends almost entirely on a narrow group of compounds that have been in use for several decades:

- Diminazene aceturate: Introduced more than 65 years ago, it remains the primary treatment for surra and bovine babesiosis.
- Imidocarb dipropionate: Used for over 40 years, it continues to be the main therapeutic option for *Babesia* infections in cattle, small ruminants, and horses.
- Buparvaquone: Developed in the 1980s, it remains the only highly effective drug for tropical theileriosis caused by *T. annulata*.

The absence of alternative drug classes due to limited research investment, regulatory barriers, or restricted commercial interest creates a vulnerable therapeutic landscape. Resistance to any of these compounds could have severe regional consequences. Quantitative epidemiological evidence supports the role of drug misuse as a driver of parasite persistence. Multivariate risk-factor modelling in Egypt demonstrated that irregular antiprotozoal drug use was independently associated with both babesiosis and theileriosis [41]. This provides field-level evidence that inappropriate chemotherapy practices sustain transmission and increase selection pressure for resistance.

Current animal-management practices aimed at controlling protozoan infections include vector control, husbandry interventions, and chemotherapy. However, in many systems chemotherapy remains the dominant control strategy and is often applied repeatedly and empirically. This pattern underscores the need for integrated parasite-management approaches that combine rational drug use with vector control, improved husbandry, and where feasible host genetic resistance.

Recent genomic frameworks demonstrate that resistance to *Theileria*, *Babesia*, and *Trypanosoma* is influenced by host immune genes (e.g., BoLA-DRB3, TLR4) and parasite drug-resistance genes such as *cytochrome-b* and *MDR1*, supporting a One-Health, genome-informed approach to sustainable parasite control [5].

## DISCUSSION

This review highlights a clear gradient in the strength and maturity of evidence for antiparasitic drug resistance among protozoan blood parasites affecting livestock in the MENA region. Among the three parasite groups assessed, *T. annulata* stands out as having the most robust and reproducible body of evidence supporting true drug resistance. Multiple independent studies from different countries consistently link buparvaquone treatment failure to specific, well-characterized molecular alterations particularly mutations in the *cytochrome-b* gene and *TaPIN1* [34,40]. Importantly, these markers are conserved across apicomplexan parasites and have been validated using complementary field, in vitro, and molecular approaches [20,35,37]. As a result, *T. annulata* represents a parasite for which resistance surveillance is technically feasible and scientifically justified at a regional scale.

In contrast, the situation for *T. evansi* reflects a substantial knowledge gap. While repeated treatment failure and relapse are widely reported under field conditions and supported by experimental resistance models [19,30,42], molecular mechanisms underlying trypanocide resistance in *T. evansi* remain undefined. This gap is particularly striking when contrasted with African trypanosomes such as *T. brucei* and *T. congolense*, where resistance mechanisms involving drug transporters and uptake pathways are well established [5,17]. The absence of equivalent markers for *T. evansi* severely limits the ability to distinguish true resistance from treatment failure caused by operational or biological confounders, underscoring the urgent need for genomic and transcriptomic investigations.

For *Babesia* spp., the evidence base is even more limited. Clinical studies from MENA consistently report variable or incomplete responses to imidocarb dipropionate and diminazene aceturate [42,43,44], but none have demonstrated genetic resistance mechanisms. In this context, it is essential to differentiate between functional treatment failure where drugs improve clinical signs without achieving parasitological clearance and true, genetically mediated resistance [42]. The absence of validated molecular markers for Babesia drug resistance, both regionally and globally, necessitates caution in interpretation and highlights a critical methodological gap in current surveillance efforts [5,17].

Across all parasite groups, the review identifies shared structural and behavioral drivers that strongly favor the emergence of drug resistance in the MENA region. Foremost among these is the extraordinary reliance on a small number of chemotherapeutic agents that have been in continuous use for several decades often exceeding 40–65 years [5,6,17]. Such prolonged exposure creates sustained selective pressure on parasite populations, particularly in endemic settings where reinfection is frequent.

Livestock production systems in MENA further amplify this risk. Pastoral and transhumant management practices facilitate extensive animal movement across ecological zones and national borders, increasing opportunities for the spread of less susceptible parasite strains. Empirical treatment without laboratory confirmation, inaccurate dosing, repeated retreatment, and the availability of substandard or counterfeit drugs exacerbate selection pressure. Epidemiological studies from the region demonstrate that irregular antiprotozoal drug use is independently associated with infection risk [39], reinforcing the role of poor drug stewardship as a key driver of resistance emergence. Together, these factors create a high-risk environment in which declining drug efficacy is not only plausible but expected, particularly in the absence of systematic monitoring and regulation.

The findings of this review point to several urgent priorities for both research and policy. First, there is a clear need to establish regional molecular surveillance networks, beginning with *T. annulata*, where validated resistance markers already exist [34,38]. Incorporating *cytochrome-b* and *TaPIN1* screening into reference laboratories would allow early detection and mapping of resistant genotypes. Second, standardized drug-efficacy trials are essential for *T. evansi* and *Babesia* spp., incorporating post-treatment molecular monitoring to distinguish relapses, reinfection, and true resistance [5,17]. Parallel investment in whole-genome sequencing and comparative genomics will be critical for identifying candidate resistance markers in these parasites. Third, policy interventions must prioritize rational drug use, including updated treatment guidelines, regulation of veterinary pharmaceuticals, and improved access to diagnostics [6,39]. Finally, sustainable control will require integrated parasite-management strategies that move beyond chemotherapy alone, combining vector control, improved husbandry, host genetic resistance, and where available vaccination [5]. In summary, while confirmed molecular resistance is currently best documented for *T. annulata*, the broader patterns observed across parasites indicate that the MENA region is at a pivotal point. Without coordinated surveillance, research investment, and policy reform, declining drug efficacy is likely to expand, threatening livestock health and food security across the region [5,17].
